# HCV Genotyping from NGS Short Reads and Its Application in Genotype Detection from HCV Mixed Infected Plasma

**DOI:** 10.1371/journal.pone.0122082

**Published:** 2015-04-01

**Authors:** Ping Qiu, Richard Stevens, Bo Wei, Fred Lahser, Anita Y. M. Howe, Joel A. Klappenbach, Matthew J. Marton

**Affiliations:** 1 Molecular Biomarker and Diagnostics, Merck Research Laboratories, Rahway, New Jersey, United States of America; 2 Target & Pathway Biology, Merck Research Laboratories, Boston, Massachusetts, United States of America; 3 Infectious Diseases and Clinical Virology, Merck Research Laboratories, Kenilworth, New Jersey, United States of America; Kaohsiung Medical University Hospital, Kaohsiung Medical University, TAIWAN

## Abstract

Genotyping of hepatitis C virus (HCV) plays an important role in the treatment of HCV. As new genotype-specific treatment options become available, it has become increasingly important to have accurate HCV genotype and subtype information to ensure that the most appropriate treatment regimen is selected. Most current genotyping methods are unable to detect mixed genotypes from two or more HCV infections. Next generation sequencing (NGS) allows for rapid and low cost mass sequencing of viral genomes and provides an opportunity to probe the viral population from a single host. In this paper, the possibility of using short NGS reads for direct HCV genotyping without genome assembly was evaluated. We surveyed the publicly-available genetic content of three HCV drug target regions (NS3, NS5A, NS5B) in terms of whether these genes contained genotype-specific regions that could predict genotype. Six genotypes and 38 subtypes were included in this study. An automated phylogenetic analysis based HCV genotyping method was implemented and used to assess different HCV target gene regions. Candidate regions of 250-bp each were found for all three genes that have enough genetic information to predict HCV genotypes/subtypes. Validation using public datasets shows 100% genotyping accuracy. To test whether these 250-bp regions were sufficient to identify mixed genotypes, we developed a random primer-based method to sequence HCV plasma samples containing mixtures of two HCV genotypes in different ratios. We were able to determine the genotypes without ambiguity and to quantify the ratio of the abundances of the mixed genotypes in the samples. These data provide a proof-of-concept that this random primed, NGS-based short-read genotyping approach does not need prior information about the viral population and is capable of detecting mixed viral infection.

## Introduction

About 130–170 million people are infected with HCV, with a global prevalence of infection estimated at 2%-3% [1,2]. Hepatitis C virus has a positive-sense single-stranded RNA genome of about 9.6 kb containing one long open reading frame (ORF) with untranslated regions at both ends [3]. So far, six major genotypes (HCV 1–6) have been described, each containing multiple subtypes [4,5,6]. Genotyping of the hepatitis C virus (HCV) plays an important role in the treatment of HCV because genotype determination has recently been incorporated into the treatment guidelines for HCV infections [7,8,9]. The optimal treatment options are becoming even more distinctive with the introduction of antiviral drugs that target specific viral proteins, such as the protease inhibitors (telaprevir and boceprevir), NS5A inhibitor (ledipasvir) and polymerase inhibitor (sofosbuvir).

Commercially available HCV genotyping assays include the Trugene HCV 5′NC genotyping kit (Bayer Healthcare, Berkeley, CA, USA), a semi-automated sequencing assay targeting the highly conserved 5’ UTR, and the VERSANT HCV Genotype Assay (LiPA) 2.0 (Siemens, Tarrytown, NY, USA), an automated reverse hybridization line-probe assay targeting the 5′NCR and core regions [10,11,12,13]. In 2013 the US FDA approved the Abbott m2000 RealTime HCV Genotype II assay (Abbott Molecular Inc., Des Plaines, IL, USA), which is a Polymerase Chain Reaction (PCR)-based assay targeting specific regions of the 5′NCR and the NS5B genes, and reports the genotypes as 1, 1a, 1b, 2, 3, 4, 5, and 6 [14]. However, it is known that the m2000 assay does not resolve all HCV genotypes [15] in which cases ‘indeterminate’, ‘mixed’, ‘genotype X reactivity with Y’, or only the major genotype were reported. To fully resolve the genotype, additional testing is required in 9–10% of cases.

The group of patients perhaps at the greatest risk for HCV infection is the intravenous drug users (IVDUs) population, for whom the prevalence of HCV infection may be as high as 80% and who are often infected with multiple virus clades prior to treatment [16]. It has been reported that as high as 20% of the patients experienced re-infection after therapy [17]. The extent of mixed genotype infections has been estimated between 2–7% [18,19] but is difficult to determine by currently available assays. Thus, it is clear that in order to meet the needs of the IVDU population there is a need for more robust assays that can detect multiple genotypes within the same specimen.

Massively parallel sequencing, also called Next Generation Sequencing (NGS), allows for rapid and low cost population sequencing of viral genomes and provides an opportunity to study the mutational landscape of HCV and viral evolution within a single host [20]. The most commonly used method for amplifying RNA virus prior to sequencing and other downstream applications is reverse transcription followed by PCR with primers designed to amplify a specific HCV genotype or degenerate primers to amplify multiple genotypes. Recent evaluations of biases generated in high throughput sequencing data have pinpointed the amplification step as the primary cause [21,22,23,24,25]. Therefore, a genotype-independent PCR-free NGS assay could have a clear advantage over PCR-based methods.

However, the development of such an assay is not without challenges. First, NGS reads are short. Most reads on the most common NGS platforms are 200–500 bp in length, so to determine HCV genotypes from NGS data using current genotyping methods will likely require HCV genome assembly. Yet, the assembly stage is complicated and may be ambiguous due to the higher error rate for NGS methods and the uncertainty of the type of mixed genotypes. In this study, we evaluated the possibility of using short NGS reads for direct HCV genotyping without prior genome assembly.

## Materials and Methods

### HCV reference selection and genotyping algorithm implementation

NS3, NS5A and NS5B sequences were retrieved from Los Alamos HCV database (LA HCV DB) (May 2014, http://hcv.lanl.gov/content/sequence/HCV/ToolsOutline.html). Redundant sequences that might belong to the same isolate (99% identity, >500bp) were removed. For each of the three viral genes (NS3, NS5A and NS5B), only subtypes with more than three unique full length sequences were included in the genotyping method implementation. Two random sequences for each genotype were chosen for reference phylogenetic tree construction. The rest of the sequences for each subtype were used as the validation dataset. The selected reference sequences for each subtype were manually checked to ensure different geographic origin and to avoid duplicate representation from same sources.

HCV reference tree sequences were aligned using CLUSTALW [26]. The resulting alignment was analyzed with the Phylip package (version 3.67) [27] building a neighbor-joining tree based on the Kimura two-parameter substitution model. The reliability of the tree topology was assessed through bootstrapping using 1000 replicates. For genotype prediction, each ‘test’ HCV sequence was placed in the reference tree and the phylogenetic tree was reconstructed using the procedure described above. The genotype of the ‘test’ sequence was extracted from the newly constructed tree file using nw_clade in Newick Utilities package [28]. The Newick Utilities are a suite of UNIX shell tools for processing phylogenetic trees.

### Selection of short HCV genome region for HCV genotyping (Genome Walking)

A 250-bp window with 100 bp overlaps was tiled through the NS3, NS5A and NS5B regions to identify the region of each gene with the best HCV genotype predictability. 250-bp was chosen arbitrarily to balance the average read length, richness of genetic information and computing speed for phylogenetic tree construction. For each 250-bp window, we retrieved the 250-bp fragments from each of the NS3, NS5A and NS5B sequences in the reference tree set. The reference tree was constructed using those 250-bp fragment regions. Genotypes for each of the sequences in the validation dataset were predicted using the reference tree according to the method described above. Prediction results were compared with Los Alamos HCV annotation. Prediction accuracy was calculated and compared for each 250-bp fragment.

### NGS sequencing of HCV containing plasma samples

Human plasma samples known to contain either HCV 1b or 3a were purchased from Boca Biolistics (http://bocabio.com) and mixed in ratios based on viral load resulting in 90% HCV 1b or 50% HCV 1b. The QIAamp Viral RNA Mini Kit (Qiagen, cat. #52904) was used to isolate RNA following the manufacturer’s instructions, including the addition of the supplied carrier RNA. In order to ensure the absence of host DNA, the purified RNA was re-isolated using the PicoPure RNA Isolation Kit (Thermo Fisher Scientific, cat. #KIT0204) with added DNase I treatment as recommended by the manufacturer. The SMARTer Universal Low Input RNA Kit (Clontech, cat. #634945) was used to generate cDNA following the manufacturer’s instructions except that the quantity of input RNA was 80 ng to compensate for the presence of carrier RNA in the original RNA isolation and no restriction enzyme RsaI digestion was performed after cDNA synthesis. Half of the resulting cDNA was used as input for the Nextera XT DNA Sample Preparation Kit (Illumina, cat. #FC-131-1096) using the Nextera XT Index Kit (Illumina, cat. #FC-131-1001). Illumina Nextera XT protocols were followed for library preparation with 0.6 volumes of purification Agentcourt Ampure XP beads (Beckman Coulter, cat. #A63880) to ensure a larger library size. No normalization was performed on individual libraries. Library quality was determined using a NanoDrop 1000 (Thermo Fisher Scientific) and Bioanalyzer 2100 (Agilent Technologies). Libraries were pooled, and sequencing was performed using an Illumina MiSeq sequencer with the 300x300 paired-end protocol. Fastq files were generated using the onboard MiSeq Reporter. The Illumina short reads generated in this study have been submitted to NCBI’s Short Read Archive (SRA) with study accession number SRP052549.

### Predicting HCV genotype from NGS data

NGS has a higher error rate than Sanger sequencing. We tested if our method can tolerate the NGS error rate (~1%) and predict HCV genotype from real NGS short reads. Paired-ends fastq data were merged using FLASH [29]. Reads with quality score less than 30 for more than 5% of the read length were removed for further analysis. The subsequent fastq files were further filtered by read length. Only reads with more than 250 bp in length were retained. Fastq files were converted into fasta files using fastx tool kit (fastq_to_fasta; http://hannonlab.cshl.edu/fastx_toolkit/). HCV 1b reference sequence Con1 was used to blast against the fasta file of the short reads generated from the previous step. Only blast hits that span NS3 (position 701–950), NS5A (position 1–250) or NS5B (position 101–350) were retained for genotyping analysis. We term these hits as ‘genotyping suitable reads’. All reverse reads from NGS were reversed and complemented first before genotyping. HCV genotyping on these ‘genotyping suitable reads’ was carried out using the phylogenetic based genotyping method described above.

## Results

### Construction of a HCV reference phylogenetic tree

**Fig 1:**
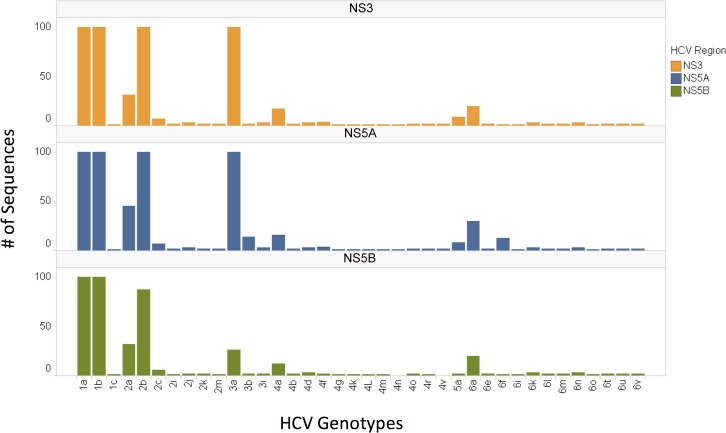
Number of sequences included in the validation set for each gene. For subtypes with >100 sequences, 100 random sequences were chosen. For the rest of the subtypes, all sequences except the sequences used in the reference set were retained for validation.

More than 20000 HCV NS3, NS5A and NS5B sequences were collected from the May 2014 version of Los Alamos HCV database. 75 total HCV subtypes were collected. Two sequences for each subtype were included in the phylogenetic based algorithm building and at least one sequence for each subtype was used for validation. Thus, only subtypes with at least 3 sequences were included in the analysis. 37 subtypes had less than 3 sequences and were excluded from the analysis and the HCV reference tree construction for NS3 and NS5A. 39 subtypes were excluded for NS5B according to the same criteria. For subtypes with a very high number of sequences, such as genotypes 1a and 1b, the number of randomly-selected sequences included in the validation dataset was capped at 100. The subtypes and the number of sequences included in the validation set were shown in Fig 1.

### Genotype predictability of different HCV regions

As an initial proof-of-concept, we tested the predictability of full length NS3. A neighbor-joining phylogenetic tree based on the Kimura two-parameter substitution model was built for the reference dataset. The genotypes of 534 out of the 540 sequences in the validation set were predicted to have the same genotype as in the Los Alamos annotation. The six sequences that showed different predicted genotypes were blasted against NCBI nt sequence database, and the annotation of the best matches was inspected manually. Table 1 lists the detailed annotation of these six sequences. Based on results from NCBI best matches and our own phylogenetic analysis on all available sequences of those HCV strains, we concluded that all six discordant sequences are either mis-annotated or ambiguous in LA HCV DB. Excluding those six sequences, 100% of the other genotype predictions for the sequences in validation set matched the Los Alamos annotations.

**Table 1:** List of entries from the validation dataset that were discordant with genotype reported in Los Alamos HCV.

Accession	Los Alamos Genotype	Predicted Genotype	Comments
EF032895	1a	1b	Annotated as 1b in original NCBI record. Wrong annotation from LA HCV DB.
KC967478	2b	2a	NCBI annotation is 2a, which suggests Los Alamos genotype is mis-annotated. Best blast hit in NCBI has no annotation and the second best hit is annotated as 2b/2a.
KC967477	2b	2a	NCBI annotation is 2a, which suggests LA genotype is mis-annotated. Data was submitted in same batch by same laboratory as KC967478.
KC967479	2c	2a	No annotation in the original NCBI submission. Best blast hit annotated as 2a, which suggests that LA annotation is mis-annotated.
JX227953	2k	2i	No subtype annotation in the original NCBI submission. Best blast hit in NCBI annotated as genotype 2. LA annotation is ambiguous. Our full gene phylogenetic analysis shows 2i
JX227952	2k	1b	Original annotation in NCBI is ambiguous and is called 2k/1b. Best blast hit in GenBank is HQ537006.1 (95% identity). HQ537006 is annotated as 2k/1b. LA annotation is ambiguous. Full gene phylogenetic analysis shows 1b

Comments provide rationale for conclusion that discordant genotype are mis-annotated by the LA HCV DB. All entries’ genotypes were confirmed by phylogenetic analysis using full gene sequences.

**Fig 2:**
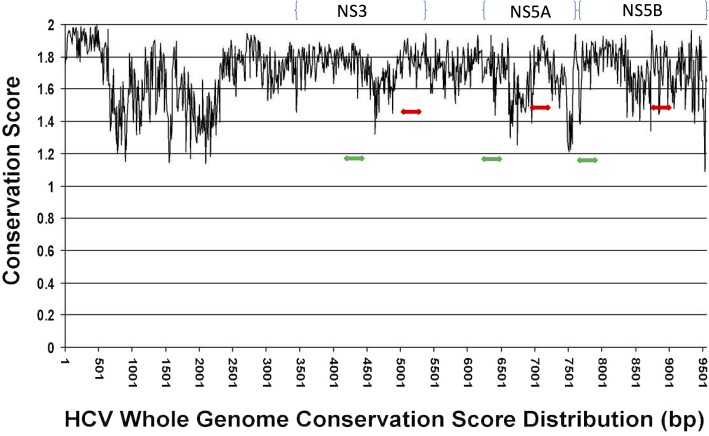
HCV genome conservation map. Entire HCV genome is displayed along the X axis. Locations of the NS3, NS5A and NS5B genes are indicated. Highest conservation score is 2 (100% conservation), as described in the Materials and Methods. Subregions with best predictive power tend to be in hypervariable regions; subregions with worst predictive power tend to be in relatively conserved regions (green arrows for good predictors; red arrows for bad predictors).

Genotype-specific genetic content may not be equally distributed across the genome. Since a smaller fragment may enable NGS short reads in genotype prediction, we next tested if a fragment of NS3 can have similar or same predictive power as full length NS3. Genome walking was done as described in Materials and Methods. Table 2 shows the predictive power of all the 250-bp fragments that were tested. Region 701 to 950 on NS3 appears to have same predictive power as the full length NS3 with 100% prediction accuracy for the validation dataset after removing the questionable Los Alamos entries. Similar analysis was performed for NS5A and NS5B. Information rich regions (1–250 bp of NS5A, 101–350 of NS5B, 601–850 of NS5B) were identified that had 100% prediction accuracy for the validation set. In general, NS5A appears to have less genetic content suitable for genotyping since there were fewer regions that resulted in genotype accuracy of > 90%, while NS5B appears to have more regions that serve to identify genotype. Although many of the subtypes tested have very few sequences due to the availability of sequences in public domain, most of the common clinical subtypes such as 1a, 1b, 2a, 2b, 3a, 4a, 5a, 6a have an adequate number of sequences. In order to investigate the general features of a good vs bad predictor region, a representative good predictor and bad predictor fragment were further mapped onto an HCV genome conservation map built previously [30]. Fig 2 shows that the good predictor regions tend to be in more divergent regions while bad predictor regions are in more conserved regions, which in general is in agreement with the conclusions published previously [31].

**Table 2:** Genotyping prediction accuracy of 250-bp windows of NS3, NS5A and NS5B subregions.

NS3				NS5A				NS5B			
Start on NS3	End on NS3	Start on HCV	Genotyping Accuracy	Start on NS5A	End on NS5A	Start on HCV	Genotyping Accuracy	Start on NS5B	End on NS5B	Start on HCV	Genotyping Accuracy
1	250	3421	90.6	1	250	6259	100.0	1	250	7603	99.8
101	350	3521	86.5	101	350	6359	94.3	101	350	7703	100.0
201	450	3621	85.4	201	450	6459	83.3	201	450	7803	98.8
301	550	3721	97.2	301	550	6559	85.8	301	550	7903	98.6
401	650	3821	99.6	401	650	6659	86.5	401	650	8003	99.5
501	750	3921	99.1	501	750	6759	99.7	501	750	8103	98.8
601	850	4021	94.6	601	850	6859	90.0	601	850	8203	100.0
701	950	4121	100.0	701	950	6959	92.2	701	950	8303	97.2
801	1050	4221	99.8	801	1050	7059	84.1	801	1050	8403	98.4
901	1150	4321	99.8	901	1150	7159	84.5	901	1150	8503	98.1
1001	1250	4421	92.3	1001	1250	7259	91.2	1001	1250	8603	99.1
1101	1350	4521	86.1	1101	1350	7359	88.4	1101	1350	8703	90.0
1201	1450	4621	89.5					1201	1450	8803	95.1
1301	1550	4721	92.3					1301	1550	8903	91.1
401	1650	4821	98.1					1401	1650	9003	81.1
1501	1750	4921	94.8					1501	1750	9103	97.9
1601	1850	5021	83.2					1601	1850	9203	97.4
								1701	1950	9303	91.4

250-bp window tiled through the NS3, NS5A and NS5B to identify the regions with the best ability to predict HCV genotype using the validation set. Each window has 100 bp overlaps. See Materials and Methods for details.

### Prediction of HCV genotypes from NGS data generated from HCV containing plasma

Currently available HCV genotyping assays were not designed to detect low frequencies of mixed genotypes. Conversely, the rapid emergence and application of NGS technology allows the cost-effective probing of virus populations at an unprecedented level of detail. However, one limitation of current NGS technology is the relatively short sequence read length it produces. If genotype prediction could be achieved without reads assembly, the reliability of the prediction may be increased dramatically. Most commercial diagnostics labs that are developing NGS-based mixed genotyping methods rely on genotype-specific PCR amplification. This step will inevitably introduce PCR bias which will likely lead to inaccurate genotyping or inaccurate quantification of mixed genotypes. To determine whether a random-primed, NGS short read approach would be sufficient to identify genotype and subtype, we performed proof-of-concept experiments with HCV-infected plasma samples.

**Fig 3:**
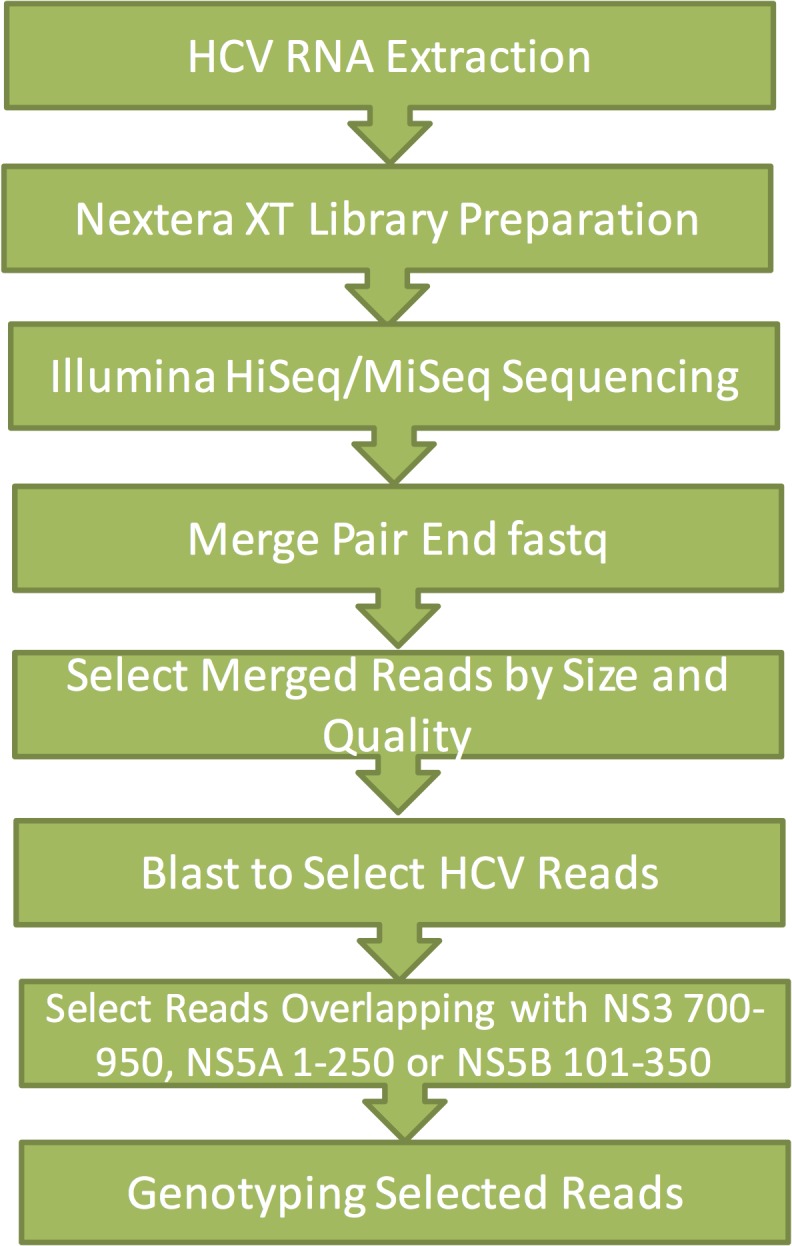
Workflow for HCV genotyping on NGS reads.

Plasma samples containing HCV genotype 1b were mixed with plasma from subjects infected with HCV genotype 3a (10^6^ IU) at a 50:50 or 90:10 ratio. HCV RNA was extracted using QIAamp Viral RNA Mini Kit, converted to cDNA and sequenced using Illumina MiSeq platform. An average of ~2 million reads were acquired for each sample; data analysis was performed as described in Materials and Methods (Fig 3). As described in Table 3, 5.2% to 9.5% of the total reads were derived from the HCV viral genome. The rest of the reads were from host DNA, RNA or host microbial nucleic acid. Using the HCV genotyping methods implemented, we were able to identify the mixed HCV genotypes from the reads covering NS3 (position 700–950), NS5A (position 1–250) or NS5B (position 101–350) that were greater than 250 bp in length. In addition, we were able to predict the percentage of each genotype and the predicted percentages were in general agreement with the expected ratio (Table 3). The observed genotype ratios predicted from replicates (S5 and S6, S3 and S4) are in good concordance. Our method is semi-quantitative and more assay development experiments are needed to determine the dynamic range and lower limit of detection (LOD) of this method using HCV samples of various genotypes with validated molecular viral quantification information if this method is to be used for clinical samples. Plasma samples with different viral loads (10^4^, 10^5^ IU) were also tested and yielded similar results. Although the number of usable NGS reads was lower in 10^4^ IU viral load sample, no false predictions were observed (data not shown). We believe that increasing the overall sequencing depth in low viral load specimens will provide enough genotyping suitable reads for mixed genotype detection.

**Table 3:** Prediction of HCV genotypes from NGS data generated from HCV containing plasma.

Sample	Total Reads	Total Combined Reads	# of Reads Q>30, Length>250	# of HCV Reads	Predictor Used	# of Reads Covering Predictor	# of GT1 Predicted	# of GT3 Predicted	Observed GT1 Freq by Predictor	Observed GT1 Freq	Expected GT1 Freq
HCV-S3	2063072	1409556	77167	4504 (5.8%)	NS3 (701–950)	70	67	3	95.7	96.6	90
HCV-S3					NS5A (1–250)	44	43	1	97.7		
HCV-S3					NS5B (101–350)	34	33	1	97.1		
HCV-S4	1218965	887188	63458	5533 (8.7%)	NS3 (701–950)	69	64	5	92.8	92.4	90
HCV-S4					NS5A (1–250)	171	159	12	93.0		
HCV-S4					NS5B (101–350)	101	92	9	91.1		
HCV-S5	2014690	1466363	73403	6999 (9.5%)	NS3 (701–950)	60	33	27	55.0	67.4	50
HCV-S5					NS5A (1–250)	159	123	36	77.4		
HCV-S5					NS5B (101–350)	124	75	49	60.5		
HCV-S6	2930073	2309270	153118	7914 (5.2%)	NS3 (701–950)	88	51	37	58.0	69.2	50
HCV-S6					NS5A (1–250)	131	96	35	73.3		
HCV-S6					NS5B (101–350)	47	37	10	78.7		

HCV plasma samples of genotype 1b and 3a were mixed as 90:10 for samples HCV-S3 and HCV-S4, and at 50:50 for samples HCV-S5 and HCV-S6.

## Discussion

In order to evaluate the possibility of using short NGS reads for direct HCV genotyping without prior genome assembly, first we surveyed the genetic diversity of the HCV NS3, NS5A and NS5B regions using publicly available HCV sequences in terms of whether the sequence was able to predict genotype. An automated phylogenetic analysis-based HCV genotyping method was implemented and used to assess different HCV genome regions. Short candidate regions were found for all three genes that have sufficient genetic information for predicting HCV genotypes/subtypes. We then applied this approach by analyzing NGS reads generated from sequencing HCV plasma containing two HCV genotypes.

One of the issues we observed during development of our genotyping method was a discrepancy between our prediction and the annotation from public domain for about 1% of the samples in our validation set. The 540-sequence validation dataset we compiled was randomly selected from LA HCV DB. For 6 sequences, our genotyping prediction conflicted with the genotypes reported in Los Alamos HCV DB. By manual inspection of their original annotations and their best blast hits in GenBank, we think it is very likely all 6 entries are either mis-annotated or ambiguous in the Los Alamos database. Our validation set is only a small portion of the publicly available HCV sequences, so it is likely many more mis-annotated HCV sequence entries exist in the public domain. Therefore, we recommended caution when using public sequences as reference sequences and suggest that genotype assignment be verified by examining different annotation sources. Sequences from the same submission generally should be avoided when selecting reference sequences to ensure diversity and to avoid bias.

HCV exists as a quasispecies [32,33]. Its high replication rate, >1000 virions per day, and the absence of a proofreading activity in the NS5B polymerase are the main factors that contribute to the emergence of mutations in the viral genome. In addition, the frequency and types of resistance associated variants (RAVs) after treatment with direct antivirals are often distinct for different subtypes. For example, the prevalence of NS3 RAVs is higher in genotype 1a than 1b. The resistance level of NS5A RAVs is 100 to 1000 fold higher in genotype 1a than genotype 1b [34,35]. Given the high degree of heterogeneity of HCV genome, treatment-caused mutation selection, the diversity of HCV subtypes and the possibility of PCR and sequencing errors, the genome assembly of HCV short reads from mixed infected samples could be very challenging and error prone. Our method bypasses genome assembly and directly identifies genotypes based on NGS short reads of 250 bp long in order to provide a more accurate readout of the real viral population in patients at risk for mixed HCV infections. The mixed infection information will help us to understand the clinical significance of RAVs in virologic failures, as well as selection of future treatment options.

We recognize that for many rare HCV subtypes in our study, the number of sequences in the validation sets is relatively small, and thus the demonstration of the predictive power for those rare subtypes might be compromised. Further validation is necessary when more sequences of these rare subtypes become available. Nevertheless, the method has been shown to be useful in the direct identification of HCV genotype from NGS short reads without the need for genotype-dependent PCR amplification, which likely introduces genotype specific bias. This genotyping method enables the simultaneous detection and identification of more than one genotype in the same plasma sample. In theory, this approach can be generally used not only for mixed HCV infection, but also for the detection of a mixed viral infection of other viral species as long as one or more species-specific information-rich fragment can be identified in their respective genomes.

One of the challenges facing HCV DAA development currently is the lack of single assay that can genotype HCV and at the same time detect and monitor drug resistant variants. Clinical samples are normally sent for genotyping first prior to resistance testing, and in some cases may even have to be assayed by a second genotyping assay when the first assay cannot identify the genotype unambiguously (e.g., the m2000 assay may report GT6 as cross-reactivity to GT1). For resistant variant analysis, the samples are then normally sequenced using Sanger sequencing, or, more recently, by NGS following genotype specific PCR amplification of the regions of interest. For rare and divergent genotypes such as genotype 6, a failed PCR amplification reaction or sequencing step is not uncommon for many samples. Genotype 6 is the most divergent genotype with more than seventy subtypes, and for many of the subtypes within genotype 6 there are a very limited number of sequences in the public databases. Designing ‘universal’ or even degenerate primers that can accommodate all genotype 6 subtypes is extremely challenging. Our method obviates the need for designing these gene- or genotype-specific primers. In addition to detecting mixed infection, another potential application of our assay is the possibility of combining genotyping and resistant variant analysis into single assay for patients infected with a single genotype. Another potential use is as a supplemental assay for extremely difficult HCV samples that fail traditional methods that rely on genotype specific information. In order to achieve this, sequencing depth needs to be increased to ensure sufficient coverage for each position of the target gene. Given that 5–10% of the total reads are from HCV genome in this study, we project that increasing the total reads 2–4 fold will provide enough sequencing depth for each position of the HCV genome (>1000 X) so that the variants can then be detected by standard variant calling pipelines while the genotype can be easily predicted from the short reads.

In conclusion, most current genotyping methods are unable to detect mixed genotypes from two or more HCV infections. Our data demonstrate a proof-of-concept that NGS short reads can be used to identify genotypes and to quantify the ratio of two genotypes in a mixed infection. The method described in this study does not involve genotype specific amplification and may be generally applicable to detecting any mixed viral infection. The method can also be used both to genotype HCV samples with rare subtypes that fail traditional genotyping and to perform variant analysis of those samples. While we anticipate assays using this approach will be pan-genotypic, additional testing is necessary to establish this. Further development and optimization of the assay is currently ongoing with different genotype mixes and a full range of viral load to support mixed genotype detection and resistant variant analysis in clinical trials.
